# Relationship between External Load and Perceptual Responses to Training in Professional Football: Effects of Quantification Method

**DOI:** 10.3390/sports7030068

**Published:** 2019-03-17

**Authors:** Vincenzo Rago, João Brito, Pedro Figueiredo, Peter Krustrup, António Rebelo

**Affiliations:** 1 Center of Research, Education, Innovation and Intervention in Sport, Faculty of Sports, University of Porto, R. Dr. Plácido da Costa 91, 4200-450 Porto, Portugal; anatal@fade.up.pt; 2 Portugal Football School, Portuguese Football Federation, Avenida das Seleções, 1495-433 Oeiras, Portugal; joao.brito@fpf.pt (J.B.); pedro.figueiredo@fpf.pt (P.F.); 3 Research Center in Sports Sciences, Health Sciences and Human Development, University Institute of Maia, Avenida Carlos de Oliveira Campos, Castêlo da Maia, 4475-690 Maia, Portugal; 4 Department of Sports Science and Clinical Biomechanics, Faculty of Health Sciences, SDU Sport and Health Sciences Cluster (SHSC), University of Southern Denmark, Campusvej 55, 5230 Odense, Denmark; pkrustrup@health.sdu.dk

**Keywords:** rating of perceived exertion, global positioning systems, physiology, team sports

## Abstract

We examined the within-player correlation between external training load (ETL) and perceptual responses to training in a professional male football team (*n* = 13 outfield players) over an eight-week competitive period. ETL was collected using 10-Hz GPS, whereas perceptual responses were accessed through rating of perceived exertion (RPE) questionnaires. Moderate-speed running (MSR), high-speed running (HSR) and sprinting were defined using arbitrary (fixed) and individualised speed zones (based on maximal aerobic speed and maximal sprinting speed). When ETL was expressed as actual distance covered within the training session, perceptual responses were moderately correlated to MSR and HSR quantified using the arbitrary method (*p* < 0.05; *r* = 0.53 to 0.59). However, the magnitude of correlations tended to increase when the individualised method was used (*p* < 0.05; *r* = 0.58 to 0.67). Distance covered by sprinting was moderately correlated to perceptual responses only when the individualised method was used (*p* < 0.05; 0.55 [0.05; 0.83] and 0.53 [0.02; 0.82]). Perceptual responses were largely correlated to the sum of distance covered within all three speed running zones, irrespective of the quantification method (*p* < 0.05; *r* = 0.58 to 0.68). When ETL was expressed as percentage of total distance covered within the training session, no significant correlations were observed (*p* > 0.05). Perceptual responses to training load seem to be better associated with ETL, when the latter is adjusted to individual fitness capacities. Moreover, reporting ETL as actual values of distance covered within the training session instead of percentual values inform better about players’ perceptual responses to training load.

## 1. Introduction

Training load monitoring is extensively employed by football practitioners to enhance performance and reduce injury risk. Common parameters for quantifying training load in professional football include rating of perceived exertion (RPE), distance covered in given speed zones derived from microtechnology incorporating global positioning systems (GPS) and exposure time to training [1]. Despite the amount of activity performed (external training load) is the main determinant of individual physiological responses (internal training load) [2], few studies have addressed the training dose–player response relationship during professional football training [3,4].

RPE is an easy, low-cost tool for measuring the perceived intensity of training sessions or matches. Despite being questionable for its subjective assessment, RPE has shown positive correlations with percentage of distance covered at high intensity [5] and with HR-based parameters such as Edwards’ training load or training impulse during practice sessions [5,6]. Moreover, Akenhead and Nassis [1] showed that RPE is one of the top five ranked variables in monitoring TL adopted in professional football. Another advantage compared to HR measurement could therefore be attributed to the fact that RPE considers both psychological and physiological factors, possibly providing a more comprehensive evaluation of TL at individual level. Recent reports have shown progressive decreases in RPE towards the end of the season, with high between-players variability (CV = 4–48%) [7]. This indicates that individual responses to training can vary markedly between players, consequently affecting coaches’ interpretation of training load data. It is not therefore surprising that practitioners show a keen interest in methods to individualise training load.

The use of GPS technologies has markedly increased over the last decade, and a wide range of metrics is available from which technical staff can be objectively informed about the training process. Arbitrary speed zones independent of players’ fitness levels seem to be commonly adopted [1,8]. However, the interpretation of arbitrary speed zones has the disadvantage of masking individual capacities, addressing TL interpretation of players’ performance rather than the load imposed by the training session on the individual. An individualised approach to adjust ETL data by players’ capacities would therefore be necessary.

Attempts to individualise ETL data have used player-dependent speed zones based on isolated fitness components, such as measures of cardiorespiratory fitness including maximal aerobic speed (MAS) [9,10]. However, the players’ MAS neither consider the players’ capacity to perform short intense actions, nor the transition from the moderate- to the high-intensity exercise domain. For instance, a powerful athlete (e.g., high maximal sprinting speed, MSS) cannot sustain high exercise intensity for long, as reflected by his intermittent-endurance capacity. Contrarily, MSS in isolation from sprint test does not account for players’ capacity to maintain high velocities for prolonged periods. Indeed, a less powerful athlete may show a comparatively higher intermittent-endurance capacity, which enables him to intensively run more frequently, entering the high-speed zones, and recover quicker. To fulfill the limitation associated to considering one fitness component only, an integrated approach is needed, combining MAS and MSS [11,12,13]. MAS is very strongly correlated with maximal oxygen uptake and, in conjunction with MSS, allows calculation of the anaerobic speed reserve (ASR) [11,14]. The combination of MAS and MSS is of importance because considering them independently to analyse ETL data would result in a misunderstanding of ETL data, neglecting the transition from an aerobic to an anaerobic regime [11]. A combined approach to quantifying ETL data that incorporates fitness data from field-based tests to estimate the players’ MAS and MSS therefore provides a more accurate definition of speed zones than a single fitness component.

To date, studies investigating the relationship between RPE and objective training load indicators in football have mainly involved subelite-level players [6,15,16]. Recently, two studies have quantified the correlation between heart rate indices and RPE in elite football players undertaking various forms of field-based football-specific training over extended periods of time [17,18]. However, currently adopted training load monitoring practices revealed a more frequent use of GPS than heart rate in professional football. It could therefore be of interest to explore the longitudinal relationship between GPS-derived indicators using different quantification methods and RPE. In addition, previous studies individualising ETL data have been predominantly conducted in young athletes [9], with scarce information available for professional players. Moreover, the aforementioned studies focused on competition only and, to the best of our knowledge, only two studies analysed individualised training ETL [13,19].

The aim of this study was therefore to examine the within-player correlation between RPE, high-speed running using arbitrary and individualised speed zones in a professional male football team.

## 2. Methods and Materials

### 2.1. Participants

During the 2016/17 season, 13 professional male outfield football players (age, height, body mass and senior experience [mean ± SD] 25.8 ± 3.5 years old, 181.5 ± 5.6 cm, 78.3 ± 5.9 kg, 7.3 ± 3.0 years) competing in Italy’s second-tier league (*Serie Bwin.it*) were regularly monitored in the context of their training routines. Sample consisted of 3 central defenders, 2 full-backs, 3 central midfielders, 2 wingers and 3 strikers. Their estimated MAS and MSS were 17.7 ± 0.6 km·h^−1^ (based on distance covered in the Yo-Yo intermittent recovery test level 1 (YYIR1) of 2289 ± 384 m) and 31.1 ± 0.9 km·h^−1^, respectively. No input was given to the technical staff throughout the data collection period. At the end of the season, the club authorised the use of a dataset for research purposes wherever anonymity was ensured. The Ethical Committee of the Faculty of Sports, University of Porto, approved and recorded the study under CEFADE.08.2018.

### 2.2. Experimental Design

Arbitrary speed zones do not take into account individual capacity, possibly resulting in incorrect interpretation of ETL. It would therefore be intuitive to evaluate the athletes’ GPS data in relation to player’ fitness profile. Differences between arbitrary and individualised speed zones have been previously documented. However, it is not known whether RPE is associated with ETL, quantified using arbitrary or individualised speed zones, and with actual (m) or relative (%) distance covered. The arbitrary method is commonly employed in professional football to quantify ETL data [8,20], whereas the use of the individualised method [12] is currently growing. Data collection was carried out over an 8-week competitive period, between January and March 2017. Individual reconditioning sessions were not included for analysis. Players participated in 42 training sessions and three friendly matches, resulting in 256 individual observations with a median of 20 training sessions per player (range: 15–25 sessions).

The present study was conducted under non-experimental conditions as the technical staff and participants did not receive any input from the research team. Training contents were described according to the typical weekly training schedule and associated daily activities. For the description, we considered each training day according to its temporal distance from match day (MD) (Table 1).

### 2.3. Procedures

#### 2.3.1. Individual Capacities as Training Load Guidance

The YYIR1 was performed on a natural grass pitch where the team usually performed training sessions. The test was chosen based on its representativeness of physical performance during official matches in professional football [21]. YYIR1 requires repeated 2 × 20 m runs (shuttles), separated by a 10 s rest period, at progressively increased speeds controlled by audio bleeps from a tape recorder [21]. The aim of the test is to perform as many shuttles as possible. The test ends when the player fail twice to reach the finish line in time. The total distance covered in the test allows to estimate MAS using a generic prediction equation as proposed by Kuipers, et al. [22].

The peak speed reached during training was assumed to be the MSS. Recent findings observed a large relationship (*r* = 0.84) and trivial bias (~0.30 km∙h^−1^) between peak speed obtained by timing gates over a 40 m sprint and peak speed obtained by GPS [23]. Additionally, it was found higher peak speeds during official matches than using timing gates for speed assessment, calling into question the use of sprint testing [23]. MSS was therefore obtained from GPS by extrapolating raw data for speed and the highest value (in km·h^−1^) recorded throughout the data collection period was retained as individual MSS. ASR was subsequently determined as the difference between MSS and MAS, and expressed in km∙h^−1^, as previously reported [12,14].

#### 2.3.2. External Training Load

ETL was monitored using unobtrusive portable 10-Hz GPS units (BT-Q1000 Ex, QStarz, Taipei, Taiwan). The mean number of satellites during data collection was 14 ± 1, and the mean horizontal dilution of position was 0.7 ± 0.1. The system used the GPS Doppler data, and distances were calculated from changes in position according to the integrated manufacturer’s proprietary algorithm, to reduce measurement error. A vest was tightly fitted to each player to place the receiver between the scapulae. The accuracy of 10-Hz GPSs has been previously examined, giving an inter-unit coefficient of variation <5% [24]. All devices were activated 15 min before data collection to allow acquisition of satellite signals in accordance with the manufacturer’s instructions. Also, to avoid inter-unit error, players wore the same GPS device for all training sessions [25]. Total distance and distance covered in each speed zone were calculated using a custom Excel spreadsheet from instantaneous raw data for time, speed and distance, and the minimum effort duration was 0.2 s. This analysis process was repeated twice, once applying global speed thresholds and once applying individual speed thresholds.

For describing ETL, three speed categories were established [moderate-speed running (MSR), high-speed running (HSR) and sprinting], and two different approaches were adopted. The arbitrary method was based on arbitrary (player-independent) speed categories: MSR, HSR and sprinting were set as 14.4–19.8 km·h^−1^, 19.9–25.1 km·h^−1^ and ≥25.2 km·h^−1^, suggested for professional male players in previous studies [8]. In the individualised method, MSR, HSR were set as 80–99.9% MAS, 100% MAS −29% ASR and ≥30% ASR [12]. In addition, total high-intensity activity (THIA) was given by the sum of MSR, HSR and sprinting. Firstly, data were reported as actual distance covered (m) within each zone using both ETL quantification methods. Secondly, to explore the possible effect of training volume, ETL was reported as a percentage of total distance covered within the training session. The technical staff decided to not use GPS during competitive matches.

#### 2.3.3. Rating of Perceived Exertion

Throughout the season, the same fitness coaches collected players’ individual RPE using Borg’s category ratio scale (CR10) after training sessions. Player RPE was collected in isolation to avoid the potential effects of peer pressure 15–30 min after each training session. This ensured that the perceived effort reflected the whole session and not the most recent exercise intensity [6]. All players were familiarised with use of the scale during the previous months. The overall training load was calculated by using session-RPE (s-RPE), that is calculated multiplying the RPE score (in arbitrary units) by the individual training duration (in min) [26].

### 2.4. Statistical Analyses

Descriptive data were reported as mean ± standard deviation (SD). To characterize the inter-session variability, the coefficient of variation (CV) for THIA was calculated dividing the between-session SD by the mean and then multiply by 100. Within-participant correlations were calculated between RPE-derived parameters (RPE and s-RPE) and ETL [27]. In repeated-measures studies, it is important to quantify within-subject correlations by modelling the longitudinal dataset as a whole and reducing the variation between subjects. This approach quantifies the correlation and associated 95% confidence intervals (95% CI) between a covariate and outcome while taking into account the within-participant nature of the study design, based on the correct degrees of freedom. The magnitudes of correlation were qualitatively interpreted using the following criteria: trivial (*r* ≤ 0.1), small (*r* = 0.1–0.3), moderate (*r* = 0.3–0.5), large (*r* = 0.5–0.7), very large (*r* = 0.7–0.9) and almost perfect (*r* ≥ 0.9) [28]. When 95% confidence intervals overlapped positive and negative values, the effect was deemed to be unclear. Otherwise, the correlation was interpreted as the observed magnitude. Significance was set at *p* < 0.05. Data analysis was performed using Statistical Package for Social Science statistical software (version 23, IBM SPSS Statistics, Chicago, IL, USA).

## 3. Results

### 3.1. Overview of Training Load

**Figure 1:**
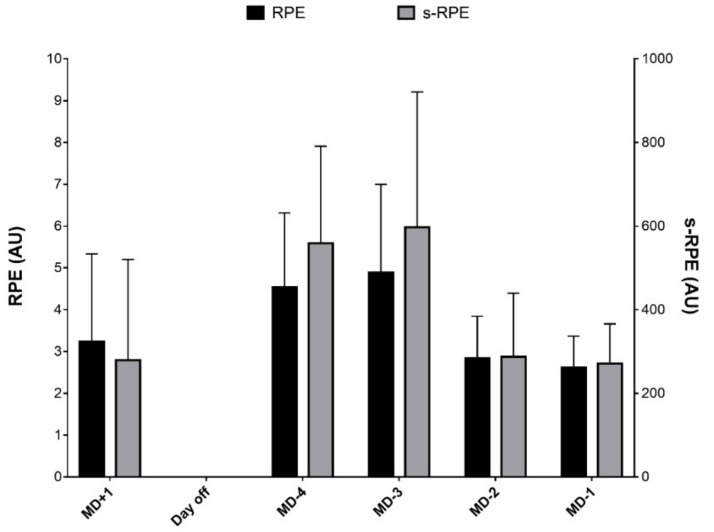
Weekly RPE and s-RPE throughout a typical weekly microcycle during a competitive period in professional football (*n* = 256 training observations). AU = arbitrary units, RPE = rating of perceived exertion, s-RPE = session rating of perceived exertion.

**Figure 2:**
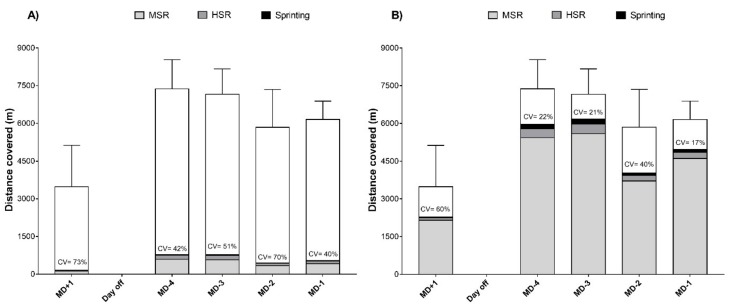
Weekly external training load during a competitive period in professional football (*n* = 256 training observations) quantified using (**A**) arbitrary speed zones and (**B**) individualised speed zones. HSR, high-speed running; MSR, moderate-speed running. The top of the bars indicates total distance covered. The coefficient of variation (CV) refers to the inter-session variability of total high-intensity activity (MSR + HSR + Sprinting).

Average exposure time, total distance covered within the training session, RPE and s-RPE were (Mean ± SD) 105 ± 23 min, 6384 ± 1593 m, 3.6 ± 1.8 AU and 411.0 ± 266.9 AU. A description of the weekly RPE is reported in Figure 1. The actual distance covered within training session was: MSR (arbitrary and individualised), 531 ± 318 m and 5000 ± 1767 m; HSR, 178 ± 173 m and 332 ± 208 m; and sprinting, 25 ± 49 m and 190 ± 183 m (Figure 2A). The inter-session variability in the actual THIA ranged between 40% and 73%. The percentage of distance covered in a given speed was: MSR (arbitrary and individualised), 8.1 ± 4.3% and 78.7 ± 18.8%; HSR, 2.7 ± 2.6% and 5.0 ± 2.8%; sprinting, 0.4 ± 0.7% and 3.0 ± 2.9% (Figure 2B). The inter-session variability in the percentage of THIA ranged between 22% and 60%.

### 3.2. Associations between External Training Load and Perceptual Responses to Training

When ETL was expressed as actual distance covered, the RPE and s-RPE were moderately correlated to MSR and HSR quantified using the arbitrary method (*p* < 0.05; *r* = 0.53 to 0.59). However, the magnitude of correlations tended to increase when the individualised method was used (*p* < 0.05; *r* = 0.58 to 0.67). Distance covered by sprinting was moderately correlated to RPE and s-RPE, only when the individualised method was used (*p* < 0.05; 0.55 [0.05; 0.83] and 0.53 [0.02; 0.82], respectively). Both RPE parameters were largely correlated to THIA, irrespective of the quantification method adopted (*p* < 0.05; *r* = 0.58 to 0.68). When ETL was expressed as percentage of total distance covered within the training session, no significant correlations were observed (*p* > 0.05). A detailed description of the relationship between RPE parameters and ETL is reported in Table 2.

## 4. Discussion

The aim of this study was to examine the within-player correlation between RPE and ETL using arbitrary and individualised speed zones in professional football. RPE and s-RPE seem to be better associated with ETL when speed zones are determined individually than when arbitrary speed zones are used, with special emphasis on sprinting distance. Notwithstanding, these correlations were only observed when ETL was expressed as actual values of distance covered, which suggests that quantifying distance covered in a given speed as portion of total distance covered does not inform about the players’ perceptual responses to training load.

In the present research, RPE-derived parameters showed moderate correlations with distance covered by MSR and THSR (*r* = 0.56 to 0.67), calculated using both arbitrary and individualised methods. This is supported by previous studies performed across football players from different competitive standards. For instance, Gaudino, Iaia, Strudwick, Hawkins, Alberti, Atkinson and Gregson [4] showed that the actual distance covered at >14.4 km·h^−1^, the number of impacts and the number of accelerations were largely correlated to s-RPE (*r* = 0.61 to 0.72) throughout the English Premier League season. Moreover, findings in young players showed moderate correlations between distance covered at >13 km·h^−1^ and RPE (*r* = 0.43 to 0.54) [5]. In addition, player load was found to be strongly associated with RPE across various small-sided game formats in semi-professional male Spanish players (*r* = 0.70) [16]. Regarding HSR, large correlations were observed only between RPE-derived parameters and individualised speed (100% MAS–29% ASR), possibly indicating that knowledge of individual MAS and ASR may better assist in understanding the effect of ETL on players’ perception of training load than the use of arbitrary speed zones (14.4 to 19.8 km·h^−1^). In this context, recent findings in ~17-year-old football players showed that training time spent above MAS would solely improve the dose-response relationship between ETL and cardiorespiratory adaptations [29]. Indeed, the authors showed that time spent above MAS was better associated (*R*^2^ = 0.59) with changes in MAS than time spent above 17 km·h^−1^ [29]. In addition, irrespective of the ETL quantification method adopted, perceptual responses to training were not correlated to distance covered by sprinting. This possibly indicates that cardiorespiratory fitness (e.g., MAS) is a stronger contributor of training load and associated perceptual responses, rather than explosive capacity (e.g., MSS). Recent match-analysis reports in female football players that found a greater number of repeated sprint sequences using a threshold of 90% MSS compared to an arbitrary one of 20 km·h^−1^ [30]. Moreover, a recent study on ~17-year-old football players reported large associations between sprinting time (≥30% ASR) and cardiorespiratory adaptations, compared to unclear associations when using an arbitrary threshold of 21 km·h^−1^ [29].

The aforementioned correlations were not observed when ETL was expressed as portion of total distance covered within the training session. This possibly indicates that perceptions of training intensity (RPE) and load (s-RPE) are affected by training volume. Under controlled situations, RPE responses increase linearly over time, reaching maximal values at the end of exercise [31,32,33]. However, football training is characterized by intermittent and spontaneous activities, and by increasing session/bout duration, the players may adopt a pacing strategy decreasing exercise intensity, or simply as a consequence of fatigue [34]. Reports of amateur adult soccer players revealed that increasing bout duration from 2 to 6 min resulted in a decreased mean HR during 3 vs. 3 small-sided games [34]. Moreover, after 2 vs. 2 to 4 vs. 4 SSGs consisting of continuous regime (1 bouts × 6 min duration) and short duration (6 bouts × 2 min) higher RPE were observed, than after 3 bouts × 4 min, or long 2 bouts  ×  6 min [35].

The usefulness of individualised speed zones has been previously documented in direct laboratory measurements (e.g., respiratory compensation threshold) that showed that a combination of MAS and MSS was more accurate in relation to gold standard measurements of heart rate deflection point and MSS, than a single fitness component per se [11]. Nonetheless, ETL is still nowadays commonly collected and interpreted on an absolute basis, expressing players’ workload as distances covered using player-independent speed zones [1]. Arbitrary speed thresholds allow comparison of physical performance between players or within the same individual player over time. However, the arbitrary approach masks the relative intensity imposed on the individual player [11]. This gap is furtherly emphasised when simultaneously monitoring athletes of different fitness levels and maturity offset [36].

Nonetheless, significant limitations of the current investigation must be pointed out. Firstly, the players could not have attained their actual MSS during training. Indeed, previous studies have evaluated MSS using the best (lower time) 10 m stretch across 40 m straight sprint [12,30] by means of timing gates, which is considered the gold standard for measuring sprint performance. Recent reports in professional players showed a moderate correlation between MSS attained during training sessions and the measure of 40 m sprint time (*r* = 0.52) [37]. An almost perfect correlation coefficient (*r* ≥ 0.90) has been suggested to be necessary for the validity of measurements [38]. Secondly, we analysed a relatively small sample of training sessions (*n* = 268 training observations) compared to previous research adopting similar designs. For instance, a recent study by Abbott, Brickley and Smeeton [13] compared ETL using arbitrary and individualised thresholds over 645 training sessions. Although a large-scale analysis such as one or multiple seasons would be necessary to generalise findings, the results of our study support previous findings showing that a significant amount of high-intensity activity is accounted for when considering individualised speed zones [11,13]. Thirdly, neither arbitrary nor individualised speed zones account for the transition between speed zones, represented by accelerations and decelerations. This is of utmost importance given the significant physiological strain (e.g., increased blood lactate concentrations, mean heart rate and perception of effort, compared to constant-speed running) associated with changing speed [39].

In summary, this is the first study to quantify the within-subject correlations between rating of perceived exertion and two different approaches to quantifying external training load. We found that testing professional male football players individually can add value to external training load monitoring and interpretation of data collected during training sessions. Specifically, using data from fitness tests to individualise speed zones appears to improve the relationship between player perceptual response to training load and running-based training load, especially in relation to sprinting distance.

## 5. Conclusions

The present research analysed the within-subject correlations between external training load running-based parameters and RPE-derived parameters in professional football players. The magnitude of the relationships between external training load and RPE parameters appear to slightly strengthen when ETL are adjusted to individual fitness capacities, with special emphasis on cardiorespiratory fitness. However, when ETL are quantified as portion of total distance covered within the training session, these relationships were unclear. Practitioners should consider ETL as actual values of distance covered within the training sessions adjusted for individual speed being more representative of perceptual responses to training, rather than percentage of total distance.

## Figures and Tables

**Table 1:** Weekly training characterization in an Italian *Serie Bwin.it* team.

Characteristic	MD + 1	MD-5 (Day Off)	MD-4	MD-3	MD-2	MD-1	MD
Day	Sunday	Monday	Tuesday	Wednesday	Thursday	Friday	Saturday
Duration	75 ± 30 min		120 ± 16 min	118 ± 15 min	98 ± 30 min	101 ± 12 min	90
Time	Morning			Bidaily	Afternoon	Afternoon	Afternoon
Warm-up	Static stretching for starting players; dynamic stretching for non-starting players.		Technical skills warm-up	Dynamic stretching	Technical skills	Dynamic stretching	
Main contents	Recovery training for starting players; small-sided games (ball possession) and cardiorespiratory endurance training for non-starting players		(1) Team tactics (e.g., 10 vs. 10 full-sized game); (2) Cardiorespiratory endurance exercises (e.g., interval training); (3) Continuous regime small-sided games (pitch was commonly goal to halfway line as length and touchline to touchline as width)	(1) Complex training (morning); (2) Intermittent-regime small-sided games (commonly ball-possession without goalkeepers) with reduced pitch sizes (e.g., 3 vs. 3 to 5 vs. 5).	(1) Team tactics (e.g., 11 vs. 11 emphasising specific and expected game situations); (2) free-kicks	(1) Corners and free-kicks; (2) Pre-match activation (e.g., skipping and short sprints)	

MD, match day; non-starters, players who participated less than ≤45 min in the game of the previous day; starters, players who participated at least 45 min in the game-time of the previous day.

**Table 2:** Relationship between rating of perceived exertion parameters and distance covered in each speed zone over an eight-week competitive period in professional male football players (*n* = 256 training observations).

Variable			Moderate-Speed Running			High-Speed Running			Sprinting			Total High-Intensity Activity		
			*p*	*r* (95% CIs)	Descriptor	*p*	*r* (95% CIs)	Descriptor	*p*	*r* (95% CIs)	Descriptor	*p*	*r* (95% CIs)	Descriptor
Training volume (m)	RPE	Arbitrary	0.002	0.56 (0.14; 0.84)	Moderate	0.011	0.55 (0.05; 0.83)	Moderate	0.184	0.34 (−0.25; 0.75)	Unclear	0.005	0.58 (0.04; 0.85)	Large
		Individualised	0.005	0.58 (0.04; 0.85)	Moderate	0.005	0.58 (0.04; 0.85)	Large	0.011	0.55 (0.05; 0.83)	Moderate	0.002	0.61 (0.08; 0.86)	Large
	s-RPE	Arbitrary	0.004	0.59 (0.04; 0.85)	Moderate	0.014	0.53 (0.02; 0.82)	Moderate	0.221	0.32 (−0.28; 0.74)	Unclear	0.004	0.59 (0.04; 0.85)	Large
		Individualised	<0.001	0.67 (0.18; 0.89)	Large	0.003	0.60 (0.07; 0.86)	Large	0.014	0.53 (0.02; 0.82)	Moderate	<0.001	0.68 (0.20; 0.89)	Large
Training intensity (%TD)	RPE	Arbitrary	0.050	0.46 (−0.12; 0.80)	Unclear	0.063	0.44 (−0.14; 0.79)	Unclear	0.273	0.29 (−0.31; 0.72)	Unclear	0.036	0.48 (−0.09; 0.81)	Unclear
		Individualised	0.094	0.41 (−0.18; 0.78)	Unclear	0.036	0.48 (−0.09; 0.81)	Unclear	0.063	0.44 (−0.14; 0.79)	Unclear	0.050	0.46 (−0.12; 0.80)	Unclear
	s-RPE	Arbitrary	0.083	0.42 (−0.17; 0.78)	Unclear	0.115	0.39 (−0.20; 0.77)	Unclear	0.327	0.26 (−0.33; 0.70)	Unclear	0.073	0.43 (−0.15; 0.79)	Unclear
		Individualised	0.115	0.39 (−0.20; 0.77)	Unclear	0.063	0.44 (−0.14; 0.79)	Unclear	0.125	0.38 (−0.21; 0.76)	Unclear	0.073	0.43 (−0.15; 0.79)	Unclear

TD, total distance covered; bold letters denote significant correlations.
